# Evidence for the formation of fused aromatic ring structures in an organic soil profile in the early diagenesis

**DOI:** 10.1038/s41598-023-39181-8

**Published:** 2023-07-31

**Authors:** Jeewan Gamage, Paul Voroney, Adam Gillespie, Andy Lo, James Longstaffe

**Affiliations:** 1 School of Environmental Sciences, University of Guelph, 50 Stone Road East, Guelph, ON N1G 2W1 Canada; 2 Advanced Analysis Centre, NMR Centre, University of Guelph, 50 Stone Road East, Guelph, ON N1G 2W1 Canada

**Keywords:** Biogeochemistry, Climate sciences, Environmental sciences

## Abstract

The presence of fused aromatic ring (FAR) structures in soil define the stability of the recalcitrant soil organic matter (RSOM). FAR are important skeletal features in RSOM that contribute to its extended residence time. During the early diagenesis, FAR structures are formed through condensation and polymerization of biomolecules produced during plant residue and microbial product decay. Molecular level characterization of the RSOM extracted from an organic soil profile gives important insights into the formation of FAR. Advanced solid-state ^13^C nuclear magnetic resonance (NMR) spectroscopy, including recoupled long-range C–H dipolar dephasing experiments on extracted humic acids (HA) showed that they contain diagenetically formed FAR different from charcoal and lignin. Peaks characteristic of FAR are observed at all depths in the soil profile, with a greater prevalence observed in the HA extracts from the clay soil layer at the bottom. In the clay soil layer, 78% of the aromatic carbon was non-protonated, and this was 2.2-fold higher than the topsoil. These data further strengthen our understanding of the humification process that could occur in early diagenesis and help explain the importance of incorporating diagenesis as an important phenomenon for long-term carbon sequestration in soil.

## Introduction

Fused aromatic ring (FAR) structures are central to understanding the nature of soil organic matter (SOM) because of their dominant role in long-term organic carbon (C) sequestration. FAR are resistant to microbial decomposition in soils due to their interior covalently-bonded carbon of fused aromatic ring structures^1–3^. Among the chemical reaction pathways contributing to FAR formation in soil, early diagenesis/humification processes have not received much attention. Previous research has focused on pathways such as charred residues of open biomass burning, atmospheric deposition of aerosols, and incorporation of biochar into the soil^1^. Hydroxyl radical-initiated oxidation of the aromatic molecules derived from lignin can form FAR^4^ during humification through condensation and polymerization reactions^5^. Kogel-Knabner et al.^6^ observed that during lignin degradation, non-protonated aromatic carbons (i.e., more condensed aromatic structures) increased while phenolic- and aromatic methoxyl-C decreased. Apart from lignin, certain lipids and compounds containing polymethylenic molecules (waxes, suberins) can also contribute to the formation of condensed aromatic molecules^7^.

Research using electrospray ionization coupled to Fourier transform ion cyclotron resonance mass spectrometry (ESI-FTICR-MS) has shown that humic acids (HAs) are composed of lignin fragments, proteinaceous materials, tannins, lipids and carbohydrates^8^, which can be considered as the potential precursors of FAR. The research has highlighted the prevalence of condensed aromatic molecules containing oxygenated functional groups, N- or S-containing groups, and carboxyl-containing alicyclic molecules (CCAM), which also link to the carboxyl-rich alicyclic molecules (CRAM) in the DOM^8^. Previous studies showed the formation of FAR and CRAM is possible from terrestrial dissolved organic matter and lignin in the presence of hydroxyl radicals (⋅OH) and iron in laboratory experiments^4,9^. Chen et al.^1^, for the first time in a wheat straw incubation experiment, identified the formation of FAR in relatively high concentrations after 360 days. Higuchi et al.^10^ described oxidation reactions that involve hydroxylation and ring opening of lignin to produce unsaturated aliphatic acids, hydroxylated carboxylic acids and hydroxylated muconic acids and that synthesize FAR and CRAM structures through polymerization and condensation reactions. Microorganisms, enzymes and free radicals significantly affect FAR and CRAM formation^11^ in the early diagenesis process. Prevailing research evidence suggest that ⋅OH are a key factor in creating the chemical bonds that lead to cross-linkages and condensation reactions to form FAR^12^. The ⋅OH needed in these condensation reactions can be formed through oxidation of reduced iron (Fe(II) by dissolved oxygen (O_2_) (Fenton-type reactions)^13,14^ and enzymatic reactions (oxidative reactions by peroxidases and phenoloxidases)^15,16^. Page et al.^17^ studied the formation of ⋅OH over a wide soil moisture gradient and showed that ⋅OH formation increased with increasing concentrations of dissolved organic matter (DOM) and iron^17^. Trusiak et al.^18^ observed that an average precipitation event of 4 mm would produce 200 μmol ⋅OH m^−2^ per day while under waterlogged conditions, would still produce 60 μmol ⋅OH m^−2^ per day. Therefore, it can be assumed that the reactants needed to form condensed aromatics/FAR are present in soils as well as the microbial processes that produce them.

Due to the complexity of the constituents of SOM, the processes underlying the synthesis of FAR are challenging to unveil. Chen et al.^1^ used advanced solid-state ^13^C nuclear magnetic resonance (NMR) spectroscopy, i.e. recoupled long-range C-H dipolar dephasing, exchange with protonated and non-protonated spectral editing (EXPANSE) and dipolar-dephased double-quantum/single-quantum (DQ/SQ) spectroscopy to demonstrated the formation of FAR. Jokic et al.^19^ and Hardie et al.^20^ described abiotic formation of humic acids through an integrated polyphenol-Maillard reaction pathway. Their research showed the formation of aromatic polymers resulting from the oxidation of glucose, glycine, pyrogallol and resorcinol by birnessite. From a biochemical perspective, extracellular oxidoreductase enzymes such as tyrosinase, laccase and peroxidases can catalyze the oxidative coupling of phenolic compounds derived from lignins, tannins and plant and microbial secondary synthesized metabolites^16^ to form more condensed aromatic structures. In summary, the research supports the hypothesis that the primary and secondary biomolecules released during decomposition undergo early diagenesis reactions that yield condensed aromatic structures and aliphatic fragments that are more resistant to further decomposition.

Numerous investigations of the stability of SOM have shown that the recalcitrancy of SOM increases with depth in the soil profile^21–23^. While the formation of FAR has been studied intensively in the laboratory^8,11,17^, their formation in natural soil environments has not received sufficient research attention. In this study, we investigated molecular-level changes in the organic matter of an organic soil profile with a focus on FAR. Muck soils can possess an Oh horizon at depth in the profile, where the organic matter is at an advanced stage of decomposition (more humified), and beneath that, a gleyed mineral soil with low hydraulic conductivity^24,25^. The microenvironment, aerobic at the top and partially anaerobic in the deeper soil layers, makes it an ideal soil profile to study the formation and stabilization of FAR. The carbon-rich aerobic environment support decomposition processes and provides the opportunity to synthesize biomolecules with the involvement of ⋅OH; a retarded microbial activity in anaerobic environments contributes to the preservation of the formed FAR and CRAM structures. Because of this unique soil environment, we hypothesize that studying the nature of the organic matter in a muck soil profile can elucidate the formation and stabilization of FAR. We focused our study on quantifying the non-protonated aromatic fraction directly associated with FAR of the organic matter using a series of solid-state NMR experiments and linking that to the formation of FAR. We applied recoupled long-range C–H dipolar dephasing experiments to estimate the cluster size differences and to distinguish FAR formed through diagenesis from lignin and pyrogenic condensed aromatic molecules.

## Materials and methods

### Soil sampling and properties

Muck soil from a depth sequence of topsoil (TS) 0–20 cm, mid soil (MS) 30–70 cm, and clay sediment (CS) 90 cm were collected from the Muck Crops Research Station/ Holland Marsh-Bradford (44°02′29.6″N 79°35′55.4″W)^26^. This area has a mean annual rainfall of 691.1 mm and a mean annual temperature of 7 °C ranging from 27 °C in July to − 12 °C on January^26^. Drainage tiles have been installed 1.2 m deep and 10 m apart to control the water table. There is no evidence or documentation indicating that fire or biochar application has occurred at the study site^18^. Prior to the establishment (1946) of the research station, the area was a marshland.

### Soil profile description

**Plate 1:**
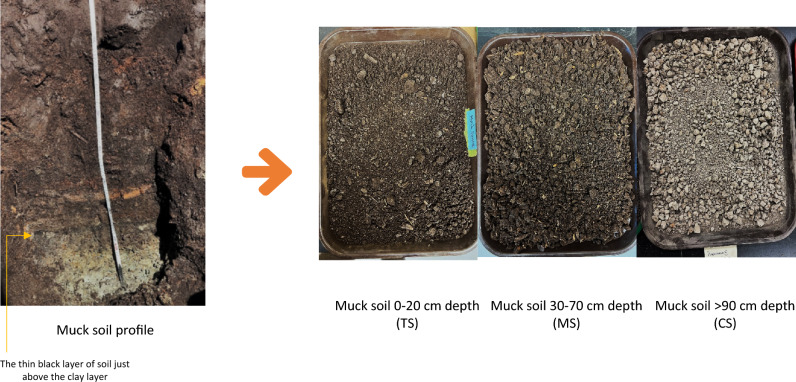
Muck soil profile from the Muck Crops Research Station, Bradford, Canada.

The soil is classified as a Humic Folisol^27^/Saprists^28^; three horizons were identified in the soil profile according to their hydrology^29^. The topsoil layer, which is the 0–20 cm (TS), was aerobic black-coloured and contained few identifiable plant remains. At the 30–70 cm depth (MS), a few large pieces of fibrous plant remains were identified. The organic soil was underlain at the 90 cm depth by a gleyish clay-mineral soil layer (Plate 1). Above the clay-mineral layer, we observed a thin (2 cm) dark black soil layer (please refer to Table 1 for the properties of the soil profile).

**Table 1:** Percent of total carbon, inorganic carbon, organic carbon, total nitrogen, ash, C/N ratio and pH of samples taken from the profile of a muck soil.

	Muck surface (0–20 cm)	Muck soil (30–70 cm)	Muck clay (> 90 cm)
pH	5.7	5.7	6.9
TC %	46.4	44.3	2.2
SIC %	0.9	1.6	0.1
SOC %	45.4	42.7	2.0
N%	3.1	2.7	0.2
C:N	14.5	16.0	10.7
Ash %	14.2	11.5	92.3

### Soil preparation and humic acid extraction

The soil was air-dried and passed through a 250 µm sieve to obtain a fine soil fraction. Particulate organic residues that accumulated during sieving were carefully removed by handpicking. Humic acid extraction was carried out according to the modified method described by Stevenson^30^ and Swift^31^. All the steps in the HA extraction procedure were performed under an atmosphere of argon in the glovebox. In brief, the acid pre-treated samples from three soil layers (each soil layer 25 g × 3 tubes) were neutralized with 1 M NaOH to pH 7 and then 0.1 M NaOH was added to give a solution to soil ratio of 10:1. The suspension was shaken at 110 rpm for 4 h and the alkaline suspension allowed to settle overnight. The supernatant was collected by centrifugation (2400 g for 20 min at 25 °C) and then acidified with 1 M HCl to pH ~ 1.2 with constant stirring followed by left standing for overnight. The HA precipitate was separated by centrifugation (2400 g for 20 min 25 °C). The HA precipitate from CS layer was suspended in a 50 mL of 0.1 M HCl: 0.2 M HF solution overnight to dissolve any associated silicate material to lower the ash content below 1%. All the precipitates were transferred to 1000 Da molecular weight cut-off (MWCO) dialysis tubes to retain the maximum amounts of HA in the dialysis process. HA from three depths was dialyzed with Millipore nanopure water until the EC reached 1 μs^−1^. After dialysis, samples were freeze-dried and used for the NMR spectroscopy experiments.

### NMR spectroscopy

**Figure 1:**
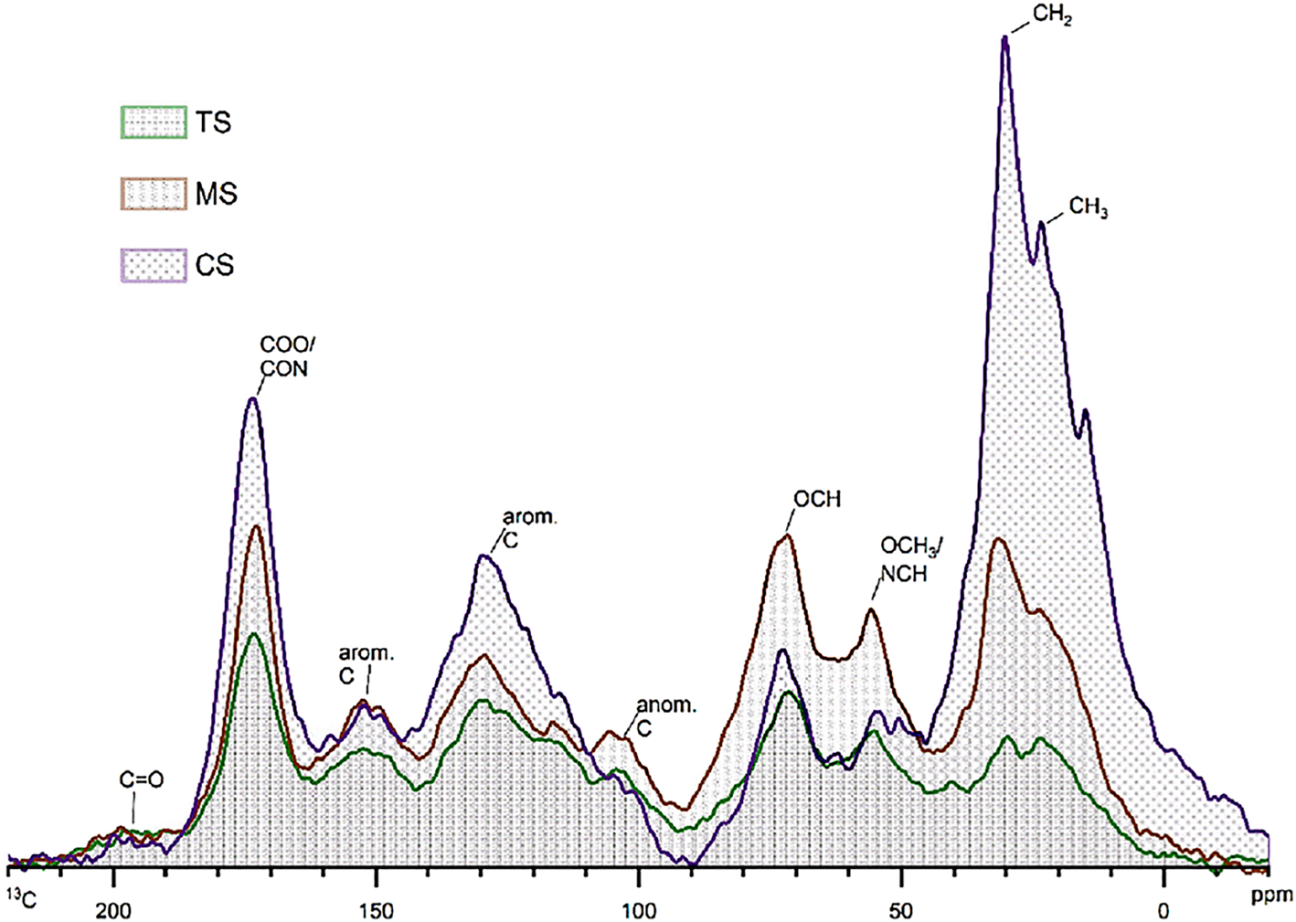
Quantitative ^13^C direct polarization magic angle spinning (DPMAS) nuclear magnetic resonance spectra at a spinning speed of 12.5 kHz of HA extracted from muck topsoil (TS) 0–20 cm, HA extracted from muck soil at 30–70 cm depth (MS), HA extracted from muck soil from mineral soil layer at > 90 cm depth (CS).

We followed the NMR experiments as described by Mao et al.^32^ to calculate the aromatic and non-protonated aromatic carbon fractions in humic acids. The NMR experiments were conducted using a 600 MHz Bruker Avance III spectrometer at 150 MHz for ^13^C, available at the Advance Analysis Centre, University of Guelph. In brief, we conducted direct polarization magic angle spinning (DPMAS) experiments spinning at 12.5 kHz (Fig. 1). The recycle delay (RD) was calculated by running a cross-polarization (CP)/T1/total sideband suppression (TOSS), which ensured that all carbon sites would be relaxed to < 5% within the RD (calculated RD were 5–150 s). Dipolar dephased direct polarization magic angle spinning experiments (dd-DPMAS) were conducted to quantify the non-protonated aromatics. A dipolar dephasing time of 67 µs was selected, which resulted in the dephasing of the magnetization of protonated carbons under the C-H dipolar coupling and retained signals of non-protonated carbons and highly mobile CH_3_^32^. We used a CSA (chemical shift anisotropy) filtered CPTOSS magic angle spinning (CPTOSS-MAS) experiment to separate alkyl O-C-O carbons from aromatic carbons. The non-protonated alkyl O-C-O carbons were separated from aromatic carbons using CSA-filtered dipolar dephased-CPTOSS MAS.

**Figure 2:**
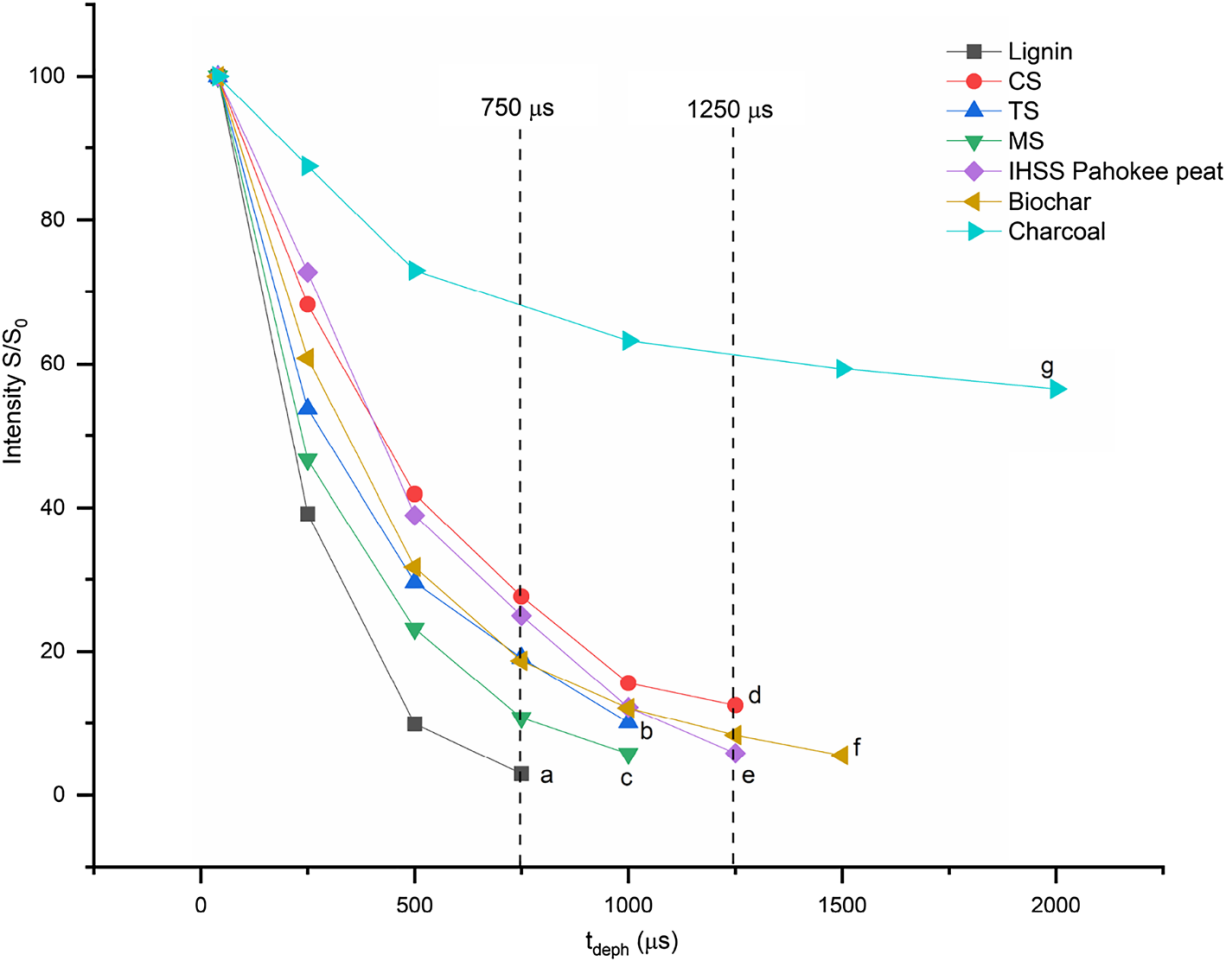
Long-range dipolar dephasing curves for the HAs extracted from muck soil profile at (b) topsoil (0–20 cm) (c) mid soil (30–70 cm) (d) clay soil (> 90 cm) HA. For reference (a) softwood lignin (e) IHSS peat HA standard (Pahokee peat) (f) softwood biochar pyrolyzed at 550 °C (g) softwood charcoal from SigmaAldrich are also shown. Y-axis is the percentage of aromatic signals (107–142 ppm) after long-range recoupled dipolar dephasing (lrdd) of the indicated durations t_deph_; x-axis is dephasing time in µs.

The FAR cluster sizes were determined using recoupled long-range H-C dipolar dephasing (lrdd) experiments^33,34^ (Fig. 2). To increase the efficiency of detecting non-protonated carbon from individual aromatic rings, we used a recoupled ^1^H-^13^C dipolar dephasing technique^33^ with direct polarization/total sideband suppression (DPTOSS). For comparison we used Pahokee peat HS standard from the International Humic Substances Society (IHSS), soft milled wood lignin obtained from MeadWestvaco Corporation (USA), softwood biochar slow-pyrolyzed under 550 °C and wood charcoal (Sigma Aldrich) (Fig. 2).

### Calculation of the degree of aromaticity in muck soil organic matter

An overlap of anomeric C (O-C-O) and aromatic C in the 90.1–120 ppm range of the ^13^C NMR spectrum can result in an overestimation of both the aromatic C fraction and the non-protonated aromatic fraction. Here, the proportion of anomeric C (90.1–120 ppm) was obtained through integration of the ^13^C CSA filtered spectrum, which essentially selected all sp^3^-hybridized carbon. For the non-protonated anomeric C, the integral for the region 90.1–120 ppm was obtained from the dd-CSA CPTOSS spectrum. This double filtering technique selected only non-protonated, mobile sp^3^ hybridized C resonances. A detailed description of the method was reported in Mao et al.^35^ and Mao et al.^36^. F_a_ which is the proportion of aromatic carbon and F_aN_, which is the proportion of non-protonated aromatic C, were calculated as follows. In brief, F_a_ was calculated using integrals from 90.1 to 142 ppm (mostly aromatic carbon), aromatic-ether carbon (142–162 ppm) and integral of the total spectrum (350 to − 50 ppm) in the DPMAS spectra. F_a_ was corrected for the overlapping alkyl signals using integrals obtain for sp^3^ hybridized O-C carbons (carbohydrates) (60–90.1 ppm) and sp^3^ hybridized O-C-O carbons (anomerics) (90.1–120 ppm) in the CSA filtered CPMAS spectra. F_aN_ was calculated using integrals as above in the dd-DPMAS and dd-CSA filtered CPMAS spectra. Correction for spinning sidebands was done using an analytical simulation program (Wsolids)^37^.

By following this approach, we were able to accurately determine the proportions of aromatic C and non-protonated aromatic C from the total C. (Please refer to Table 2 for the NMR peak assignments). Integrations obtained from aromatic C (sp^2^) and anomeric C from CSA filtered spectrum were used for the calculation of sp^2^ to sp^3^ ratios.

**Table 2:** Carbon species and chemical shifts via ^13^C NMR.

Chemical shift	Molecular fragments: carbon species
0–47	Unsubstituted saturated alkyl carbons (C)
47–60	Alkyl C substituted by oxygen and nitrogen atoms
60–110	Alkyl C singly bonded to one oxygen atom (such as ring C in carbohydrates) and alkyl C bonded to two oxygen atoms (such as anomeric C of carbohydrates)
110–144	Proton and alkyl substituted aromatic C
144–164	Oxygen substituted aromatic C
164–183	Carboxyl, ester, and amide C
183–190	Quinone C

## Results

### Proportions of protonated and non-protonated aromatic C in muck soil organic matter

The muck TS had the highest percentage of aromatic-C (36.2%), which decreased with depth in the profile. The lowest proportion of aromatic C was recorded for the CS sample, which was 22.9% of the total organic C. HA from the MS recorded the highest proportion of non-protonated aromatic C, which was 29%. HA extracted from CS, and TS layers were comprised of 17.8% and 12.8% of non-protonated aromatic C, respectively. Examination of the sp^2^ to sp^3^ ratios showed that the HAs in both TS and CS had similar ratios (1.13 and 1.07, respectively). The ratio between the non-protonated aromatic C fraction (F_aN_) to the aromatic C fraction (F_a_) in CS indicated that 77.8% of the organic C was non-protonated C and derived from FAR (refer to Table 3 for the comparison of calculated C fractions between three soil layers). Since our main focus of the study was determining the proportions of non-protonated aromatic C we did not calculate the proportions of some of the other functional groups which require additional NMR experiments (please refer to Mao et al.^38^ for further information).

**Table 3:** Proportions of carbon fractions calculated from the ^13^C NMR spectra of the HA extracted from the three soil layers and reference materials (IHSS Pahokee peat and Lignin).

Fraction	IHSS Pahokee peat	Lignin	Muck surface 0–20 cm (TS)	Muck 30–70 cm (MS)	Muck 90 cm (CS)
Non-phenolic aromatic carbon fraction	42.7	51.8	25.6	21.2	15.7
Phenolic and aromatic ether carbon fraction	7.7	9.6	3.8	10.6	5.1
other non-protonated aromatic carbon fractions	24.8	14.1	8.5	19.1	12.7
O-C-O fraction	3.0	2.7	5.5	5.6	7
Non-protonated O-C-O fraction	0.5	0.2	0.8	0.4	0.1
Carbohydrate fraction	8.4	8.9	12.8	16.5	6.5
Aromatic C (f_a_)	55.6	68.2	36.2	30.7	22.9
Non-protonated aromatic C (F_aN_)	33.6	23.7	12.8	29	17.8
F_aN_/F_a_	60.4	34.7	35.5	94.5	77.8
1 − F_aN_	0.66	0.76	0.87	0.71	0.82
sp^2^ to sp^3^ ratio	0.8	1.2	1.13	0.55	1.07

### Proportions of alkyl C, O-alkyl C and carboxyl C fractions

The proportion of carbohydrates (mainly O-alkyl carbons) in the total organic-C was calculated from the ratio of the integral of 60–90 ppm to the total integral of the DPMAS spectrum. HA extracted from the MS had the highest proportion of carbohydrates (16.5%), and the CS had the lowest (6.5%). We observed an increase of proportion in the carboxylic region (187.3–162 ppm) from TS to CS in the soil profile (TS-7.7, MS-9.9, CS-17.2) indicating that the degree of humification was highest in the CS (Fig. 1).

### Degree of aromatic condensation

Two complementary approaches were used to estimate the degree of aromatic condensation. Recoupled long-range dipolar dephasing NMR is related to the spatial relationship between C and protons^1^. The larger the aromatic ring cluster size, the less signal attenuation will be observed due to protons being, on average, further from C. Previous studies have shown that monoaromatic signals from structures such as lignin were attenuated to less than 3% of their original signal strength after a dephasing time of 0.86 ms, whereas signals from more condensed aromatics like charcoal were less attenuated^33^. Interestingly, aromatic signals from HAs extracted from CS still had 20% of their signal strength after a dephasing time of 1 ms, which indicates the presence of more condensed aromatic structures than lignin. Increasing the dephasing time to 2.5 ms results in 5% of the original signal being retained and suggesting the presence of FAR in these CS HA extracts. This pattern was similar to that of wood charcoal, which is well-known for containing highly condensed aromatic compounds. The aromatic signals of the HA extracted from the MS were attenuated to ~ 6% after a dephasing time of 1 ms, compared to ~ 9% for the HA from TS at 1 ms (Fig. 2). This difference suggests that aromatic signals for TS HA have comparatively more condensed aromatic structures compared to the MS HA. Further, this may indicate that FAR structures in MS processed further to reduce cluster sizes. The biochar sample showed the same trends as the MS HA for shorter dephasing times but shows slower signal attenuation than MS HA for longer dephasing times. The study site has no history of biochar incorporation into the soil and structural similarities between the MS HA and the biochar are likely due to this biochar being produced at low temperatures, which results in smaller cluster sizes compared to biochar produced at higher temperatures^34,39^. The differences observed in the dephasing behavior of the organic soil profile, wood charcoal, and lignin, particularly at longer dephasing times, suggest that the presence of pyrogenic carbon is unlikely in the organic soil profile.

To understand the characteristics of FAR, we compared the dephasing curves of the muck soil profile with those of the International Humic Substances Society (IHSS) Pahokee peat HS standard, soft milled wood lignin obtained from MeadWestvaco Corporation (USA), softwood biochar slow-pyrolyzed under 550 °C and wood charcoal (Sigma Aldrich). We plotted the percentage of the aromatic signal from the 107–142 region, which is mostly from aromatics, against the ^13^C^1^H dipolar dephasing time (t_deph_) (Fig. 2) because Mao et al.^33^ and Brewer et al.^34^ have shown that soils containing more fused/condensed aromatics had a slower dephasing time. In that aspect, HAs extracted from CS dephased more slowly compared to those from the TS and MS. It is apparent that the dephased curve for the HA from MS is similar to that of lignin, whereas the dephased curve for CS was more comparable to the dephased curve of wood charcoal (Fig. 2).

Based on our aromaticity calculations (Table 3), we were able to calculate 1 − F_aN_, which is indicative of the fraction of carbons along the edges of the aromatic rings^34^. 1 − F_aN_ numeral decreases with increasing aromatic ring cluster sizes. The FAR, with their aromatic ring edges attached to other C atoms, have a lower 1 − F_aN_ value compared to lignin and to more monomeric aromatics. 1 − F_aN_ values for the HAs from TS and CS were 0.87 and 0.82, respectively, and those extracted from MS had the lowest value, 0.71.

**Figure 3:**
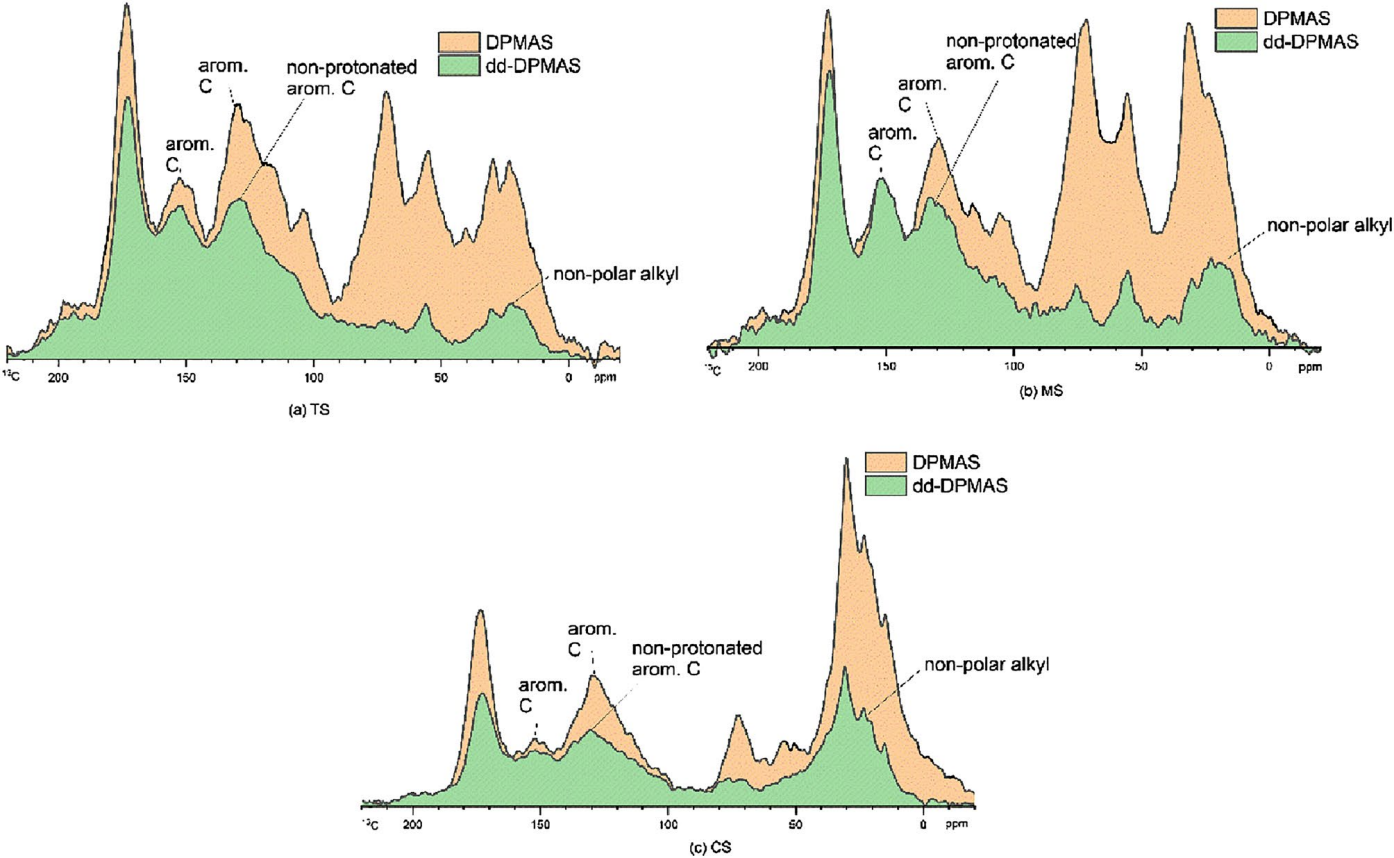
Quantitative ^13^C direct polarization magic angle spinning (DPMAS) nuclear magnetic resonance spectra at a spinning speed of 12.5 kHz and corresponding dipolar dephased (dd) time of 67 μs, sub-spectra of (a) HA extracted from muck topsoil (TS) 0–20 cm (b) HA extracted from muck soil at 30–70 cm depth (MS) (c) HA extracted from muck soil from mineral soil layer at > 90 cm (CS).

Features in the DPMAS spectra (Fig. 1) and dipolar dephased DPMAS spectra (Fig. 3) between the three soil layers showed differences in the composition and intensities of the peaks related to different functional groups. The low and broad signal at ~ 150 ppm for O-substituted aromatic C (aromatic C-O) indicates phenolic groups or aromatic C-O-CH_3_^40–42^, while a slightly higher, broad peak at ~ 130 ppm is due to the resonances of non-protonated aromatic C^1^ A peak at ~ 105 ppm was attributed to the anomeric C (double oxygen substituted alkyl C) commonly found in sugar rings of carbohydrates^43^. The peak of O-alkyl (OCH) at 72 ppm is derived from carbohydrates. The peak from OCH_3_/NCH at 55 ppm indicates either methoxy (OCH_3_) or amine groups (NCH). The peak of CH_2_ at 40 ppm also appears with a peak of CH_3_ at 22 ppm; both may occur in aliphatic chains such as lipids and waxes^36,44^. The spectrum of CS differed from others as the spectrum was dominant, with the peak at 72 ppm and 30 ppm, and the non-protonated aromatic C peak was broader and more complex. Features in the DPMAS spectra between the three soil layers showed relatively fewer changes in the 140–160 ppm range but contained significant changes in the 120–135 ppm region, particularly for the CS sample (Fig. 1). This may be due to the degradation of lignin and the formation of FAR. Moreover, it is evident that the carboxyl C content was highest at CS, which may be due to oxidation of lignin^40^. The NMR signals for samples from CS emphasize the importance of careful observation of FAR and CCAM to understand the stability of the OM at deeper depths.

## Discussion

Our study provides the first insights into the formation of FAR in the early diagenesis in a muck soil profile, focusing on changes to the condensed aromatic carbon. Typically, HA accounts for 60–85% of SOM^45^ and Chen et al.^1^ stated that 3–12% of FAR are derived from diagenetic processes. In an organic soil rich in C, diagenetic FAR can represent even more than 60% of total FAR^46,47^. Increment of the proportion of non-protonated aromatic carbon to protonated aromatic carbon indicates the enhancement of the aromatic cluster sizes^34,48–50^. The MS showed the highest proportion of non-protonated aromatic carbon and the dephasing spectra showed that the aromatic C at MS are less fused compared to CS and TS HA. This observation emphasizes the more decomposed OM illuviated to the MS and possible synthesis at CS to form more condensed aromatic structures. It was evident that a significant proportion of the aromatic C in the HA extracted from CS is more condensed compared to those extracted from TS and from MS. Examination of the long-range dipolar dephasing curves (Fig. 2) confirmed that the condensed aromatic-C in these HA extracts is clearly different from pyrogenic-C (charcoal) and lignin-C. Their formation can be explained by aromatization of lignin-derived unsaturated aliphatic acids being transformed into more condensed aromatic structures through radical polymerization or diagenesis reactions^4^. These newly formed FAR can illuviate down the soil profile and accumulate in the CS. Similar observations by Tadini et al.^51^ stated that the more labile SOM components are found in the surface horizons while the more complex and stable organic matter is leached and accumulates in the deeper Bh horizons in Amazonian Podzolic soils.

The greatest quantity of non-protonated aromatic C was recorded in the MS soil. More woody structures were seen at this depth compared to the TS. This could be due to the more chemically-resistant woody structures that exist in the MS owing to its slightly anaerobic environment that slows decomposition^52^. The proportion of non-protonated aromatic-C to the aromatic C (F_aN_/F_a_) almost doubled (35.5 to 77.8) when comparing TS and CS, suggesting that either non-protonated aromatic C is resistant to decomposition throughout the soil profile or that there is continuous formation of FAR in the soil profile that illuviate to the CS. We assume that the evidence supports the formation of FAR for the MS samples because the proportion of non-protonated aromatic C was 94% and, in the CS, it was reduced to 77.8%. If the condensed aromatic C were resistant and accumulated throughout the profile depth, we should have observed a continuous increase in the proportion of non-protonated aromatic C. This observation may also be explained as due to the high hydrophobicity of FAR and their greater retention in the organic matrix^53^. Moreover, a 1 − F_aN_ (which estimates the fraction of C along the edges of the aromatic rings^34^) of 0.87 for the TS, 0.71 for MS and 0.82 for CS is an indication that the TS had larger cluster sizes which degraded into comparatively smaller clusters, and that the clusters were larger again at the CS depth. It can be assumed that this increase in CS was due to further condensation of the aromatic C. Further support for this argument is that clay minerals are known to facilitate the formation of FAR^20^.

It is important to understand that the chemical reactions contributing to the formation of FAR are derived from the aromatization of lignin-derived unsaturated aliphatic acids through radical polymerization reactions^4^. Waggoner et al.^4^ have elaborated that in the presence of ⋅OH free radicals, unsaturated aliphatic acids can be polymerized into aromatic functional groups with carboxyl functional groups. This explanation is supported by findings that fungal enzymes can degrade lignin by hydroxylation and produce unsaturated aliphatic acids, which eventually go on in a series of polymerization reactions to produce FAR^10,54^.

A further explanation of the observed differences in proportions of non-protonated aromatics is the involvement of microorganisms in forming FAR. Previous studies have proposed that the soil microbial biomass, which is composed of complex polyketides, could be a source of FAR^55^. Under aerobic conditions, the decomposition of labile C (O-alkyl C) may contribute to the observed enrichment of FAR in MS that may have undergone further transformations in the anaerobic conditions of CS with clay minerals providing surfaces for reactions. Our argument is that this phenomenon contrasts with that of Chen et al.^1^, as they have shown that the lignin structural fragments accounted for most of the aromatic C in their incubation study of wheat straw decomposition under anaerobic conditions. We suggest that their observation could be due to the slow decomposition of lignin and in an anaerobic environment there would be the slow formation of FAR. We postulate that the intermediate chemical reactions (radical formation/catalytic reactions) required for FAR formation occur relatively slowly in an anaerobic environment, whereas in an aerobic environment, these reaction processes occur rapidly. Therefore, more anaerobic environments can better preserve FAR than aerobic environments. Another explanation is that there are differences in the microbial communities involved in the decomposition processes^52,56^ in the aerobic and anaerobic environments. We observed that the C:N ratio of the HAs extracted from CS was 10.7 (Table 1), which indicates that the organic matter which accumulated was likely of microbial origin^57^. Overall, it can be understood that the reactions of partial degradation, rearrangement and formation of recalcitrant molecular moieties occur throughout the organic soil profile in the process of early diagenesis. These products of diagenetic recalcitrant and hydrophobic FAR and CCAM are retained in the CS layer, which could exhibit longer turnover times. Chen et al.^1^ showed that the contribution of biogenically derived FAR could account for 3–12% of the total condensed aromatics produced annually in soils. We highlight that, especially in environments such as muck soils, FAR derived through early diagenesis can be the most abundant type of condensed aromatics.

## Implications and future research

Our findings further strengthen the new direction that FAR can be formed by diagenesis through a biogenic pathway which is via the decomposition of plant/microbial residues. We, for the first time, show potential chemical pathways responsible for the formation of FAR in a profile of muck soil. This knowledge further expands the theory of humification and its importance in understanding the soil organic matter decomposition processes and the transformation of microbial products to humic substances. Our findings will open a new direction in research related to soil C sequestration. An understanding of the resistant nature of FAR to microbial decomposition is essential to understanding the dynamic flow of soil organic matter in pools varying in decomposability. FAR can be associated with the microbially-resistant SOM pool possessing millennial mean residence times. Further research in the early diagenetic formation of FAR should be a major direction in examining the stable/recalcitrant SOM. Our study provides important insights into the formation of FAR in the natural environment; however, the reaction mechanisms involved in the process need to be further evaluated. Therefore, we suggest further research on molecular level reaction mechanisms in the formation of FAR, their stabilization mechanisms in the soil environment (texture, pH) and the need to reassess current SOC models to more accurately predict the dynamics of SOM. This new research will be important to further expand our knowledge of SOC cycling and the long-term potential for organic C sequestration in soils.

## Supplementary Information

Supplementary Figures.

## Data Availability

The datasets generated and analyzed during the current study are available from the corresponding author on reasonable request.
