# Supplementary material for: Evidence for the formation of fused aromatic ring structures in an organic soil profile in the early diagenesis
Source: Sci Rep. 2023 Jul 31;13:12378. doi: 10.1038/s41598-023-39181-8 (PMC10390584; doi:10.1038/s41598-023-39181-8)
Supplement: Supplementary file 1 — Supplementary Figures. [file 41598_2023_39181_MOESM1_ESM.docx]

b

a

Figure 1. Quantitative ^13^C direct polarization magic angle spinning (DPMAS) nuclear magnetic resonance spectra at a spinning speed of 12.5 kHz of (a) IHSS peat standard-Pahokee peat (b) soft milled wood lignin obtained from MeadWestvaco Corporation (USA).

d

c

Figure 2. Quantitative ^13^C long-range dipolar dephased direct polarization magic angle spinning (lrdd-DPMAS) nuclear magnetic resonance spectra at a spinning speed of 12.5 kHz and dephasing time 0.25 ms of (c) wood charcoal (Sigma Aldrich) (d) softwood biochar slow-pyrolyzed under 550 °C.
